# Toxicokinetics and tissue-specific biotransformation of modified mycotoxin zearalenone-14-glucoside (ZEN-14-G) in broilers following oral administration

**DOI:** 10.1016/j.psj.2026.106445

**Published:** 2026-01-13

**Authors:** Okasha Hamada, Haojian Sun, Xue Pan, Decheng Suo, Xia Fan, Zhigang Song

**Affiliations:** a Key Laboratory of Efficient Utilization of Non-Grain Feed Resources, College of Animal Science and Technology, Shandong Agricultural University, Taian, Shandong 271018, China; b Animal Production Department, Faculty of Agriculture, Benha University, Moshtohor 13736, Egypt; c Institute of Quality Standard and Testing Technology for Agro-Products, Chinese Academy of Agricultural Sciences, Beijing 100081, China

**Keywords:** Broilers, Modified mycotoxins, Toxicokinetic, UHPLC-Q/TOF-MS, Zearalenone-14-glucoside

## Abstract

Zearalenone-14-glucoside (ZEN-14-G), a major modified mycotoxin, has attracted considerable attention due to its potential to convert back into free zearalenone (ZEN), thereby posing toxicological risks to animals and humans. Given the limited toxicokinetic data available, this study examined the absorption, distribution, and elimination of ZEN-14-G in Arbor Acres broiler chicks following oral gavage at a dose of 0.5 mg/kg body weight to assess its potential health risks. Blood and tissue samples were collected at 0, 0.5, 1, 6, and 12 h after administration. Plasma concentrations of ZEN-14-G and its primary metabolite, ZEN, were quantified using a validated UHPLC-Q/TOF-MS method. Pharmacokinetic parameters were derived via non-compartmental analysis. The results demonstrated rapid *in vivo* hydrolysis of ZEN-14-G to ZEN. After oral administration, ZEN-14-G reached peak plasma concentration (Cmax) rapidly. It exhibited a short elimination half-life (T½), a limited apparent volume of distribution (Vd/F), and low total plasma clearance (CL/F). Notably, ZEN was detected in various tissues, with the highest concentrations observed in the liver, glandular stomach, and pectoral muscle. In summary, these findings underscore the important role of biotransformation in ZEN-14-G metabolism and provide key insights for assessing the health risks associated with broiler exposure to glucoside-modified mycotoxins.

## Introduction

Mycotoxins are fungal secondary metabolites that contaminate agricultural commodities, posing risks to both animal feed and human food supplies (Gurikar et al., 2023). Among the numerous mycotoxins, ZEN is commonly found in cereal grains (Gab-Allah et al., 2023; Qu et al., 2024) and poses significant health threats to livestock and consumers. However, emerging research has identified the existence of modified mycotoxins, including Zearalenone-14-glucoside, a conjugated form of the parent mycotoxin (Freire and Sant’Ana, 2018; Bryła et al., 2018a; Tan et al., 2023).

Although ZEN-14-G is not routinely monitored in standard regulatory surveys, accumulating evidence confirms its widespread presence in cereals, feed ingredients, and compound feeds. Surveys of naturally contaminated maize, wheat, barley, and oats consistently report frequent co‑occurrence of ZEN-14-G with ZEN, with detection frequencies often comparable to those of the parent toxin and co‑occurrence rates reaching up to 80% (De Boevre et al., 2012; Huang et al., 2014; Tebele et al., 2020). In most cases, ZEN-14-G is found at lower concentrations than ZEN, typically representing approximately 5–50% of the corresponding ZEN level; however, comparable or even higher proportions have been occasionally reported, depending on crop species, geographical origin, and environmental conditions (De Boevre et al., 2012; Berthiller et al., 2013). These findings indicate that ZEN-14-G may contribute substantially to the total ZEN‑related exposure burden in animal feeds (EFSA, 2016).

The toxicokinetics of modified mycotoxins, such as ZEN-14-G, have attracted increasing interest due to their significant implications for animal health and food safety. While the parent mycotoxin ZEN has been extensively studied (Osselaere et al., 2013; Tan et al., 2023; Qu et al., 2024), the biotransformation pathways and tissue distribution of its conjugated metabolites in poultry, particularly in broiler chicks, remain poorly characterized.

From a toxicological perspective, the relevance of ZEN-14-G is further underscored by its demonstrated potential for hydrolysis and biotransformation *in vivo*. Following oral exposure, ZEN-14-G can undergo gastrointestinal or microbial hydrolysis, release free ZEN and thus serve as a concealed source of the parent estrogenic mycotoxin (Dall’Erta et al., 2013; Dellafiora et al., 2016). Consequently, dietary exposure to ZEN-14-G may indirectly increase systemic exposure to ZEN, complicating risk assessments that rely solely on measurements of the parent compound (EFSA, 2016). Given its frequent co-occurrence with ZEN, its variable yet sometimes substantial contribution to total ZEN equivalents, and its potential for *in vivo* conversion, characterizing the toxicokinetics and tissue distribution of ZEN-14-G is essential for realistic exposure and health risk assessment (Yang et al., 2018).

Broiler chicks, a key component of the poultry industry, are highly susceptible to mycotoxin exposure due to their dependence on compounded feed (Okasha et al., 2024). Poultry exhibit particular sensitivity to ZEN toxicity, followed by ruminants-a difference likely attributable to variations in absorption kinetics, metabolic pathways, tissue distribution, and excretion mechanisms across different animal species (Qu et al., 2024). The process of masking, which converts ZEN into its modified form ZEN-14-G, adds complexity to toxicokinetic studies aimed at understanding the absorption (A), distribution (D), metabolism (M), and elimination (E) of this compound *in vivo*. Specifically, investigating the reconversion of modified mycotoxins to their parent forms in various tissues of broiler chicks after oral administration represents a critical research area with important implications for animal health and food safety. Feed contains a mixture of metabolites with differing toxicities, leading to the occurrence of ZEN (Lorenz et al., 2019; Rogowska et al., 2019). The major metabolites of ZEN include α-ZOL, β-ZOL, α-ZAL, and β-ZAL, which constitute its primary biotransformation products (Pałubicki et al., 2021). Avian species demonstrate notable tolerance to ZEN, which may be linked to the endogenous estrogenic environment typical of poultry physiology (Liu et al., 2020b).

Upon ingestion by the host organism, ZEN is rapidly and extensively absorbed through the gastrointestinal tract and subsequently distributed to specific target organs (Rai et al., 2020). Researchers have detected both zearalenone and its metabolites in various poultry tissues and excreta, including internal organs (liver, kidney, intestine), systemic circulation (blood), deposition sites (muscle), and excretory products (Buranatragool et al., 2015; Hort et al., 2020). ZEN and its biotransformation products exhibit estrogenic activity by competing with estradiol for receptor binding. This competition results in changes to the weight and morphology of reproductive organs, contributing to reproductive disorders in animals (Yuan et al., 2022). Furthermore, the pharmacokinetic profile of ZEN has been characterized in various livestock species (broilers, turkeys, and laying hens) using different administration routes, including oral ingestion and intravenous injection (Osselaere et al., 2013; Buranatragool et al., 2015; Devreese et al., 2015). Previous toxicokinetic studies have shown that the time to maximum concentration (Tmax) and the terminal elimination half-life (T½) following oral administration in poultry are shorter than those in pigs and rats, respectively (Shin et al., 2009; Schelstraete et al., 2020). Consequently, the toxicokinetic profile of zearalenone directly influences species-specific susceptibility to both the parent toxin and its masked derivatives. This susceptibility stems from variability in pharmacokinetic processing (ADME), which determines target tissue exposure and the resulting toxicological effects (Zhang et al., 2020). Evaluating these parameters is therefore essential for assessing the adverse effects of exposure to ZEN and its biotransformation products.

Understanding the toxicokinetics of masked mycotoxins in broiler chicks is essential for evaluating their absorption, distribution, metabolism, and elimination (ADME) in avian species (Schelstraete et al., 2020). Previous studies have indicated that masked mycotoxins can be enzymatically hydrolyzed in the gastrointestinal tract, releasing the parent mycotoxins and potentially enhancing their toxicological impact (Lu et al., 2020; Ekwomadu et al., 2021; Smaoui et al., 2023). However, the extent and efficiency of this conversion in broiler chicks are still not well understood (EFSA, 2017; Bryła et al., 2018b). The toxicokinetics and tissue-specific distribution of modified mycotoxins, particularly ZEN-14-G, remain poorly characterized in broiler chickens following oral exposure. Currently, comprehensive data on the absorption, metabolism, and organ accumulation patterns of these modified mycotoxins in poultry are lacking in the literature.

Based on these occurrence data and toxicological considerations, the exposure dose used in this study was selected to represent a controlled, worst-case yet biologically relevant scenario, in line with previous toxicokinetic studies on modified mycotoxins (EFSA, 2016; Yang et al., 2018). This approach enables a robust evaluation of the absorption, biotransformation, tissue distribution, and excretion of ZEN-14-G and its metabolites, without aiming to replicate typical dietary intake levels. Such a design facilitates a meaningful interpretation of the toxicokinetic behavior of ZEN-14-G and supports its integration into future exposure and risk assessment frameworks.

This study quantified the toxicokinetic parameters of ZEN-14-G in commercial broiler chicks following oral administration, with particular focus on its biotransformation to free ZEN in key gastrointestinal and metabolic tissues, including the liver, proventriculus (glandular stomach), and pectoral muscle. Using ultrahigh-performance liquid chromatography-quadrupole time-of-flight mass spectrometry (UHPLC-QTOF-MS), we investigated the toxicokinetic profile of this modified mycotoxin, encompassing its absorption, tissue distribution, and metabolic conversion. The findings clarify the bioavailability and tissue-specific accumulation patterns of ZEN-14-G and its metabolites, thereby supporting an evidence-based risk assessment of poultry-derived food products.

## Materials and methods

### Toxins and reagents

ZEN-14-G and ZEN were obtained from Shanghai ZZBIO Co., Ltd. (Shanghai, China). All other chemicals and reagents were of HPLC MS analytical grade (Merck, Darmstadt, Germany). Water was purified using a UPHN® series ultra‑pure water system (Sichuan Youpu Ultra‑Pure Technology Co., Ltd.; Chengdu, China). ZEN-14-G was dissolved in cell‑culture grade ethanol/water (20:80, v/v; ethanol from Tianjin Kaitong Chemical Reagent Co., Ltd.; Tianjin, China). Physiological saline used for oral administration was also of cell‑culture grade.

### Animals

One hundred one-day-old male broiler chicks (average body weight 43.10 ± 0.37 g) were obtained from a commercial hatchery (Liaocheng Hekangyuan Animal Husbandry Co., Ltd.; Liaocheng, China). The birds were acclimatized for one week in stainless steel cages at the animal facility of the Faculty of Animal Science and Technology, Shandong Agricultural University. They were fed a standard commercial diet and provided drinking water ad libitum. The animal study protocol was reviewed and approved by the Institutional Animal Care and Use Committee of the Faculty of Animal Science and Technology, Shandong Agricultural University (Approval No. SDAUA-2025-164).

### Experimental design

After acclimatization, fifty-four one-week-old male Arbor Acres broiler chicks were randomly selected and weighed (169.59 ± 2.73 g). They were allocated into three groups: a control group (*n* = 6) and two treatment groups (*n* = 24 each). Following a standardized 6 h fast with free access to water, all birds received their respective treatments via oral gavage.

The first treatment group (*n* = 24) received ZEN-14-G orally at 0.5 mg/kg body weight. This dose was selected based on previous experimental studies (Osselaere et al., 2013; Tardieu et al., 2020), which established its ability to elicit measurable physiological and biochemical responses without inducing acute toxicity, making it suitable for modeling chronic low-level exposure in poultry. The dose calculation considered the average daily feed intake of broilers (29.14 ± 3.69 g feed/kg BW).

To validate the experimental model and confirm endpoint sensitivity, the remaining 24 chicks were assigned to a positive control group. This group received the ethanol/water vehicle (20:80, v/v) orally, with a final ethanol volume of 0.814 mL. The ethanol concentration was minimized and kept consistent across all relevant groups, and the administration volume was standardized to preclude any independent effects on absorption or metabolism.

Blood samples were collected from the jugular vein into heparinized tubes at 0 (pre-dose), 0.5, 1-, 6-, and 12 h post-dosing. Plasma was separated by centrifugation at 1968 × g for 15 min at 4 °C and stored at −80 °C until analysis.

Following ZEN-14-G administration, birds were euthanized at designated time points. Tissue samples (liver, glandular stomach, and pectoral muscle) were collected at 0.5, 1-, 6-, and 12 h post-treatment (*n* = 6 per time point). Tissues were snap-frozen in liquid nitrogen and stored at −80 °C until analysis.

### Sample preparation

Plasma samples were thawed to room temperature, while liver, glandular stomach, and pectoral muscle tissues were mechanically homogenized. Precisely measured aliquots (0.5 mL of plasma, 0.01 g of glandular stomach, and 0.5 g each of liver and pectoral muscle) were transferred into separate 10 mL centrifuge tubes. Then, 5 mL of a 0.2 M solution (pH 3.0) prepared in acetonitrile/water (90:10, v/v) was added to each sample. After vortexing for 30 min, the mixtures were centrifuged at 10,000 rpm for 5 min. From each sample, 3 mL of the supernatant (from plasma, liver, stomach, or muscle) was transferred to a new 10 mL centrifuge tube, followed by the addition of 100 mg of a multi‑adsorbent clean‑up material and 500 mg of anhydrous magnesium sulfate. The tubes were vortexed for 30 s and centrifuged at 10,000 rpm for 2 min. The supernatants were then transferred to fresh tubes and evaporated to dryness under a nitrogen stream at 40 °C. The residues were reconstituted in 0.5 mL of acetonitrile/water (90:10, v/v) containing 0.1% formic acid, vortexed for 30 s, and after an additional 1 min of vortexing, filtered through a 0.22 µm membrane.

### Standard working solutions

Working standard solutions of ZEN-14-G and ZEN were prepared by serially diluting stock solutions (1 mg/mL) with 50% acetonitrile in water (50:50, v/v) to concentrations ranging from 0.0125 to 5 µg/mL. For the plasma calibration curves, 90 µL of blank plasma was spiked with 10 µL of each working standard, yielding final concentrations of 1.25 to 100 µg/L (seven concentration levels). For the tissue matrices (liver, glandular stomach, and pectoral muscle), 1 g of blank tissue was fortified with 100 µL of the working standard solutions to achieve the same concentration range (seven levels).

### UHPLC-Q/TOF-MS analysis

**Fig. 1:**
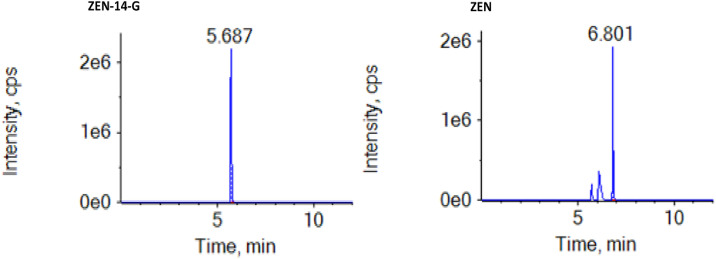
Representative UHPLC-Q/TOF-MS chromatograms of ZEN-14-G and ZEN in ESI⁺ and ESI⁻ modes.

Chromatographic separation was achieved using an ACQUITY UPLC BEH C18 column (100 mm × 3.0 mm, 1.7 µm; Waters, Framingham, MA, USA) with an injection volume of 10 µL. The mobile phases consisted of (A) water containing 1% formic acid and (B) acetonitrile (ACN), delivered at a flow rate of 0.3 mL/min under the following gradient program: 0–1 min, 10% B; 1–2 min, 10–25% B; 2–3 min, 25–30% B; 3–5 min, 90% B; 5–6 min, 10% B, followed by a 2 min re‑equilibration at initial conditions (total run time: 8 min). ZEN-14-G and ZEN were quantified using an AB Sciex 5500 UHPLC‑Q/TOF‑MS system operated in electrospray ionization (ESI) mode. Key MS parameters were: ion spray voltage 3.5 kV, desolvation gas (high‑purity nitrogen) 50 arb, collision gas 30 arb, and ion source temperature 320 °C. Additional MS settings were provided in Table 1. The method was specifically optimized and validated for the detection and quantification of ZEN-14-G and ZEN; secondary metabolites were not included in the current analytical scope. Under the optimized conditions, the method exhibited excellent chromatographic performance, with well‑resolved peaks, good symmetry, and high signal intensity (Fig. 1).

**Table 1:** Mass spectrometry conditions for the analysis of Zearalenone-14-glucoside and Zearalenone.

No.	Mycotoxin	Quantitative Ion	Qualitative Ion	Deionizer Voltage (V)	Collision Energy (Ev)
**1**	ZEN-14-G	397.1 > 317	397.1 > 317;397.1 > 175.1	100	10;15
**2**	ZEN	317.2 > 131.0	317.2 > 131.0;317.2 > 175.1	100	35;30

ZEN-14-G= Zearalenone-14-glucoside, ZEN= Zearalenone.

### Statistical analysis

Plasma concentration–time profiles and tissue residue levels of ZEN-14-G and ZEN in broiler chicks are presented as mean ± SD (*n* = 6). Toxicokinetic parameters for ZEN-14-G were determined by non‑compartmental analysis (NCA) using PK Solver software and were reported as mean ± SD (*n* = 6). All graphical representations were prepared using GraphPad Prism (version 10.4.2; GraphPad Software, Inc., San Diego, CA, USA).

## Results

### Method validation parameters

**Fig. 2:**
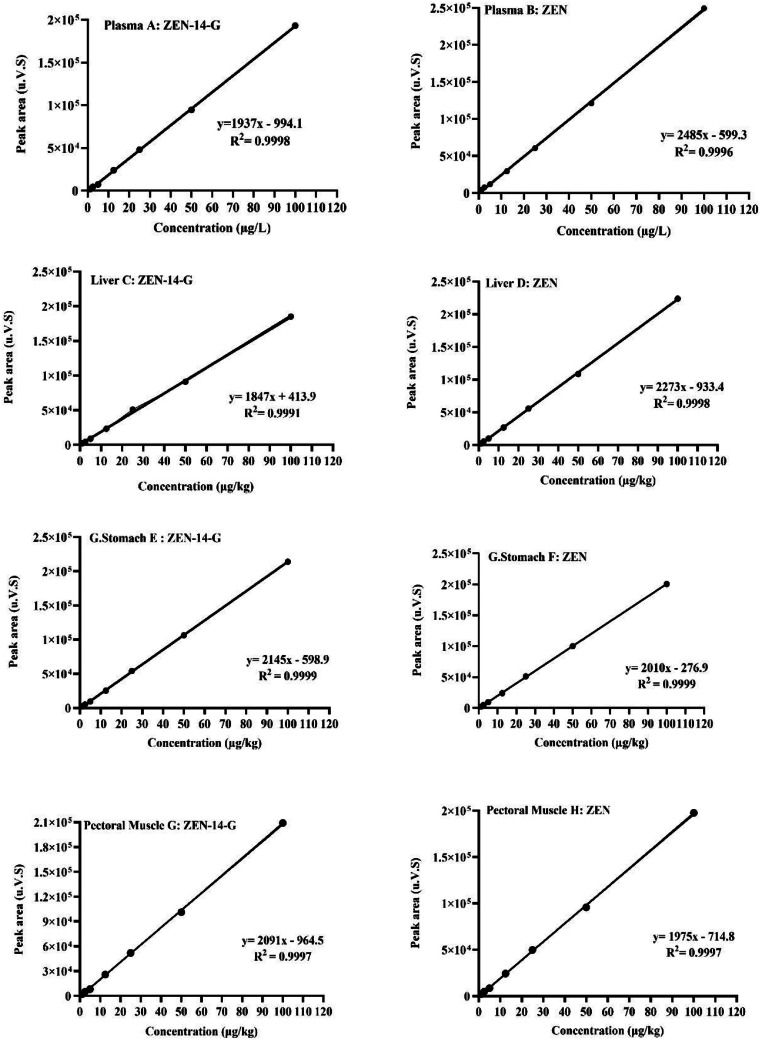
Matrix-matched calibration curves for ZEN-14-G and ZEN in (A, B) plasma (µg/L), (C, D) liver, (E, F) glandular stomach, and (G, H) pectoral muscle (µg/kg for tissues) (*n* = 3).

Calibration curves for ZEN‑14‑G and ZEN showed excellent linearity over the concentration range of 1.25–100 µg/L (Fig. 2). High correlation coefficients (R²) were obtained for both compounds’ analytes in all matrices: plasma (0.9998 for ZEN‑14‑G, 0.9996 for ZEN), liver (0.9991, 0.9999), glandular stomach (0.9999, 0.9999), and pectoral muscle (0.9997, 0.9997) (Table S1). Sensitivity was assessed by determining the limit of detection (LOD; signal‑to‑noise ratio ≥3) and limit of quantification (LOQ; signal‑to‑noise ratio ≥10). The established LOD and LOQ values varied by matrix: plasma (LOD: 1.17–1.65 µg/L; LOQ: 3.53–4.99 µg/L), liver (LOD: 1.89–2.52 µg/kg; LOQ: 3.59–7.36 µg/kg), glandular stomach (LOD: 0.73–0.76 µg/kg; LOQ: 2.21–2.30 µg/kg), and pectoral muscle (LOD: 1.43–1.49 µg/kg; LOQ: 4.32–4.52 µg/kg), as detailed in Table S1.

Recovery was calculated as the ratio of the analyte peak area in matrix‑spiked samples to that in neat standard solutions at the same concentration. Mean recoveries for ZEN‑14‑G and ZEN in plasma, liver, glandular stomach, and pectoral muscle were summarized in Table 2. Recoveries were evaluated at two spike levels (5 and 50 µg/L), representing low and high concentrations, to assess method performance across different tissue types.

**Table 2:** Determination of recovery for Zearalenone-14-glucoside and Zearalenone in plasma, liver, glandular stomach, and pectoral muscle.

Item	Spike level	Recovery (%)*			
		Plasma	Liver	Glandular stomach	Pectoral muscle
**ZEN-14-G**	**5**	85.07 ± 4.94	88.59 ± 3.29	93.64 ± 2.51	87.57 ± 2.88
	**50**	98.71 ± 3.61	96.75 ± 3.64	99.62 ± 5.00	97.74 ± 3.51
**ZEN**	**5**	98.58 ± 3.07	73.11 ± 2.71	97.78 ± 1.95	93.51 ± 2.69
	**50**	97.97 ± 3.41	85.42 ± 4.92	99.99 ± 2.89	97.63 ± 3.87

⁎ Mean±SD*, n* = 3 per concentration.

### Toxicokinetics parameters of ZEN-14-G and ZEN in broiler chicks

Following a single oral administration of ZEN-14-G (0.5 mg/kg body weight) to broiler chicks (mean body weight: 169.59 ± 2.73 g), the modified mycotoxin was absorbed into systemic circulation. Plasma concentrations of ZEN-14-G and ZEN were quantified at designated time points. As summarized in Table 3, ZEN-14-G was rapidly absorbed, reaching peak plasma levels soon after administration. It exhibited a relatively short elimination half-life, moderate systemic exposure, efficient clearance, and a limited volume of distribution-consistent with restricted tissue penetration. In contrast, ZEN showed delayed absorption, attaining peak plasma concentration at a later time point. The toxicokinetic profile of ZEN was characterized by a markedly prolonged elimination half-life, greater systemic exposure, slower clearance, and a more confined volume of distribution. These results underscore the distinct pharmacokinetic behaviors of ZEN-14-G and its metabolite ZEN.

**Table 3:** Plasma toxicokinetic parameters of ZEN-14-G and ZEN after single oral dosing in broiler chicks.

Toxicokinetic Parameter	Value of ZEN-14-G	Value of ZEN
**Body weight (g)**	167.59 ± 2.73	
**ZEN-14-G (µg/kg๒BW)**	500	
**T_max_ (h)**	0.5 ± 0.00	0.625 ± 0.125
**C_max_ (μg/ml)**	3.742 ± 0.328	7.882 ± 1.269
**T_½_ Elim (h)**	2.937 ± 0.382	10.091 ± 2.975
**AUC 0-t (μg๒h/ml)**	8.062 ± 0.432	48.768 ± 4.116
**Cl/F (ml/h/kg)**	0.045 ± 0.001	0.0066 ± 0.001
**Vd/F (ml/ kg)**	0.189 ± 0.022	0.082 ± 0.017

Pharmacokinetic parameters: T_max_ (time to peak concentration), C_max_ (peak concentration), T_½_ (elimination half-life), AUC (area under the curve), Cl (clearance), Vd (volume of distribution), F (bioavailability).

### Plasma concentrations of ZEN-14-G and ZEN

**Fig. 3:**
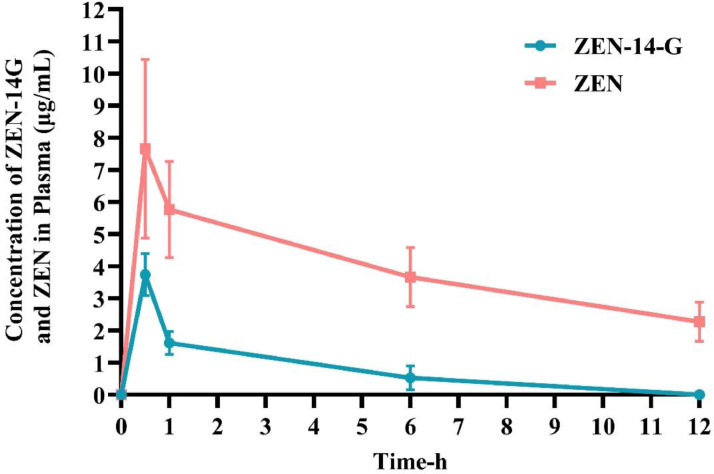
Time-dependent concentrations of ZEN-14-G and ZEN in the plasma of broilers after a single oral administration of ZEN-14-G (0.5 mg/kg BW; *n* = 6).

The plasma kinetic profile of ZEN-14-G following oral administration was presented in Fig. 3. The parent compound was detected shortly after dosing, with its concentration increasing rapidly to a peak (Cmax) before declining. In contrast, ZEN showed delayed detection but reached higher plasma levels compared to ZEN-14-G, attaining its maximum concentration (Tmax) at a later time point prior to elimination.

### Tissue detection levels of ZEN-14-G and ZEN in liver, glandular stomach, and pectoral muscle

UHPLC‑Q/TOF‑MS analysis revealed distinct tissue distribution profiles of ZEN‑14‑G and ZEN in broiler chickens following oral administration. ZEN‑14‑G was detectable in liver and glandular stomach for up to 1 h post‑dosing but was not found in pectoral muscle beyond 30 min. In contrast, ZEN showed sustained detectability, with measurable concentrations persisting for up to 12 h in all tissues examined (liver, glandular stomach, and pectoral muscle), as detailed in Table S2.

**Fig. 4:**
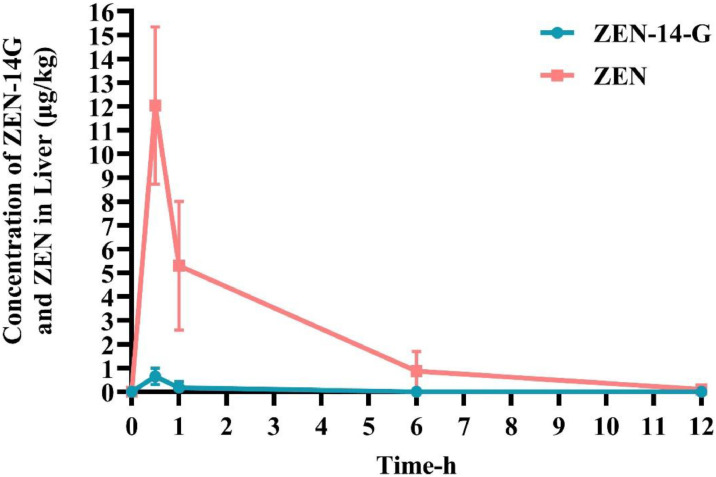
Time-dependent concentrations of ZEN-14-G and ZEN in the liver of broilers after a single oral administration of ZEN-14-G (0.5 mg/kg BW; *n* = 6).

**Fig. 5:**
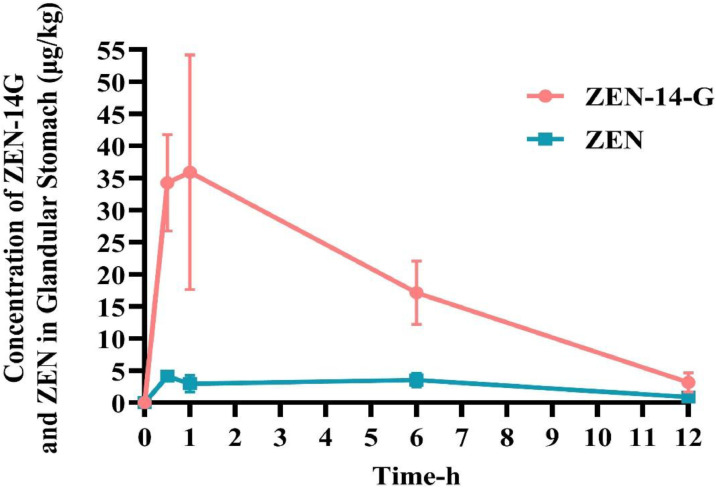
Time-dependent concentrations of ZEN-14-G and ZEN in the glandular stomach of broilers following a single oral administration of ZEN-14-G (0.5 mg/kg BW; *n* = 6).

**Fig. 6:**
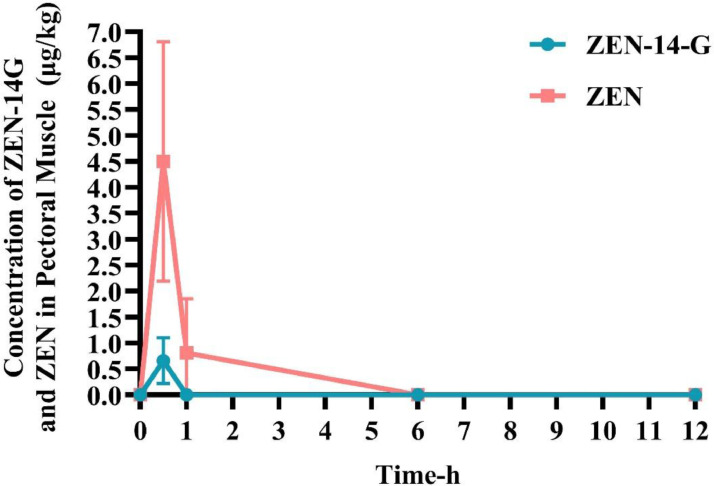
Time-dependent concentrations of ZEN-14-G and ZEN in the pectoral muscle of broiler chicks after a single oral administration of ZEN-14-G (0.5 mg/kg BW; *n* = 6).

As shown in Fig. 4, peak concentrations of ZEN‑14‑G (0.648 ± 0.345 µg/kg) and ZEN (12.037 ± 3.31 µg/kg) in liver were observed at 30 min post‑administration, followed by a gradual decline. In the glandular stomach (Fig. 5), the maximum concentration of ZEN‑14‑G (35.904 ± 18.277 µg/kg) occurred at 1 h, while that of ZEN (4.103 ± 0.895 µg/kg) was reached at 30 min, with both compounds declining progressively over the 12‑h period. In pectoral muscle (Fig. 6), peak levels of ZEN‑14‑G (0.661 ± 0.111 µg/kg) and ZEN (4.497 ± 2.307 µg/kg) were also detected at 30 min. While ZEN‑14‑G became undetectable thereafter, ZEN concentrations decreased gradually until 1 h, after which they fell below the limit of detection.

## Discussions

Bioaccumulation and biotransformation are critical processes that influence the fate of readily metabolized chemicals, potentially contributing to human toxicity. Zearalenone‑14‑glucoside (ZEN‑14‑G), a glucoside‑modified mycotoxin, has attracted global attention due to its frequent detection in food and its significant potential to revert to its toxic parent compound, ZEN (Lu et al., 2022). The toxicokinetics of ZEN (encompassing its entry into the body, absorption, distribution, metabolism, and excretion) varies across species (Qu et al., 2024). In the present study, broiler chicks were chosen as the experimental model because of their importance as a meat source for human consumption. However, the toxicokinetics of orally administered ZEN‑14‑G remain uncharacterized in broiler chicks. Therefore, this study aimed to address this knowledge gap by investigating the absorption and toxicokinetic profile of ZEN‑14‑G after a single oral gavage in broiler chicks.

Previous studies have examined the processing and breakdown of ZEN‑14‑G in rats following single doses of ZEA at 0.05, 0.75, and 10 mg/kg body weight (Sun et al., 2019; Yang et al., 2020; Lu et al., 2022). In line with earlier research, the present study employed an oral dose of 0.5 mg/kg body weight to explore the metabolism of ZEN‑14‑G in broiler chicks.

Since ZEN-14-G is a structurally modified derivative of the original mycotoxin, it retains similar absorption rate properties. Following oral administration, ZEN is swiftly and extensively absorbed in the digestive system (Zinedine et al., 2007). Absorption refers to the extent and speed at which a toxin enters the bloodstream from its administration site. Tmax, a key pharmacokinetic parameter, indicates the absorption rate by measuring the time required to reach peak plasma concentration (Guerre, 2015; Qu et al., 2024). In broiler chicks, ZEN-14-G showed rapid absorption, with a Tmax of 0.5 h, compared to ZEN, which reached Tmax at 0.625 h. In rats, following oral administration (PO) of 10 mg ZEN-14-G/kg BW, Tmax values were recorded as 0.5 h for ZEN and 0.25 h for ZEN-14-G (Lu et al., 2022). This discrepancy can be attributed to variations in animal species and administered doses across studies. After PO administration of 3 mg ZEN/kg BW to Ross 308 broiler chicks, the Tmax was 0.35 h (Devreese et al., 2015). Additionally, following a single PO administration of 1.2 mg ZEN/kg BW to broiler chickens, the peak plasma concentration was observed at 0.25 h (Buranatragool et al., 2015). Previous research in laying hens and turkeys reported Tmax values of 0.32 h and 0.97 h, respectively, after an oral dose of 3 mg/kg ZEN (Devreese et al., 2015). In pigs, Tmax was found to be 2 h following a PO dose of 1 mg ZEN/kg BW (Schelstraete et al., 2020). Overall, the Tmax of ZEN-14-G is shorter in rats than in broiler chicks and varies with dosage, whereas ZEN reaches peak concentration faster in broiler chicks than in rats, turkeys, and pigs.

The comparative pharmacokinetics of ZEN-14-G and ZEN in broiler chicks revealed that ZEN-14-G was absorbed and metabolized more rapidly than ZEN, as indicated by its earlier Tmax. This suggests that the glucoside form ZEN-14-G may undergo faster systemic uptake and subsequent metabolism, possibly due to differences in solubility, membrane transport, or enzymatic hydrolysis. The quicker absorption and metabolism of ZEN-14-G could imply an immediate but potentially shorter-lived bioavailability compared to ZEN, which may exhibit a slightly delayed but more prolonged presence in circulation. These findings highlight the importance of considering the pharmacokinetic behavior of ZEN metabolites, such as ZEN-14-G, when assessing their biological effects in broiler chicks, as their rapid turnover could influence toxicity, distribution, and overall metabolic fate. This study focused on quantifying ZEN-14-G and its parent compound, ZEN, to specifically evaluate the bioavailability and tissue distribution of this masked mycotoxin in broilers. The primary aim was to determine the contribution of ZEN-14-G to systemic ZEN exposure. Given that the toxicological relevance of ZEN-14-G stems largely from its hydrolysis to ZEN and not from further biotransformation, the formation of downstream reductive metabolites such as α- and β-zearalenol is considered secondary and unlikely to impact the reported toxicokinetic parameters. Thus, while these metabolites were outside the analytical scope, their exclusion does not affect the validity of the ZEN and ZEN-14-G kinetics, which remain the principal vectors of exposure in this model. Further research is needed to clarify the mechanisms and physiological implications of these differences, and to extend metabolic profiling to α- and β-zearalenol for a comprehensive assessment of the estrogenic burden.

Studies indicate that the oral bioavailability of zearalenone varies significantly across species. Female pigs absorb approximately 51.5% of the administered dose (Fleck et al., 2017), while broilers, laying hens, and turkey poults exhibit much lower absorption rates, ranging from 6.8% to 10.3% (Devreese et al., 2015). Given the established rapid disposition of ZEN in avian species, and preliminary evidence suggesting ZEN-14-G undergoes similarly prompt biotransformation and excretion, our sampling schedule (0–12 h) was designed specifically to characterize these early kinetic events. The observed low bioavailability of ZEN-14-G (8.89%) may result from its metabolic conversion to ZEN via deglycosylation (Sun et al., 2019). Furthermore, this rapid conversion to the parent mycotoxin (ZEN) likely accounts for its extremely low oral bioavailability (Yang et al., 2020). The apparent Vd/F reflects the relationship between the systemically absorbed dose and the measured plasma concentration. The low plasma concentration of ZEN-14-G could result from either extensive tissue distribution (a large volume of distribution) or rapid biotransformation into metabolite compounds. These experimental outcomes align with results previously documented by Sun et al. (2019). The comparatively higher volume of distribution observed for ZEN versus ZEN-14-G suggests more extensive tissue distribution of the parent compound (Fleck et al., 2017). In the current study, the similar magnitudes of distribution observed for ZEN-14-G and ZEN suggest that both compounds exhibit substantial tissue penetration, though the glucoside moiety of ZEN-14-G may moderately influence its distribution compared to the parent compound. This finding implies that while ZEN-14-G undergoes systemic absorption, its tissue distribution remains significant, possibly due to partial hydrolysis back to ZEN or independent tissue uptake mechanisms. The comparable distribution profiles highlight the need to consider both compounds in toxicity assessments, as their tissue accumulation could contribute to prolonged biological effects. CL/F quantifies the efficiency of compound elimination by measuring the virtual plasma volume that would be entirely cleared of the substance each hour. Our findings demonstrated significantly greater absorption of ZEN-14-G in plasma following oral administration, accompanied by enhanced systemic clearance compared to other administration routes (Sun et al., 2019).

While Shin et al. (2009) have thoroughly investigated the tissue distribution of ZEN, our study focused specifically on characterizing the distribution patterns of ZEN-14-G. The concentration-time profiles of ZEN-14-G and its metabolites across various tissues offer critical insights into the compound's distribution dynamics and metabolic pathways. We maintained temporal alignment with our pharmacokinetic study by using identical sampling time points for tissue analysis. ZEN-14-G showed distinct tissue-specific distribution patterns, with the highest concentrations detected in glandular stomach tissues, followed by a progressive decline over time. Secondary accumulation occurred in the liver, while substantially lower levels were observed in pectoral muscles. This finding is consistent with Lu et al. (2022), who reported that ZEN-14-G exhibited restricted tissue distribution, with quantifiable amounts confined to the stomach and absent from other examined tissues. Research suggests that ZEN-14-G primarily targets the small and large intestines, affecting enzymes from gut bacteria as well as the intestinal lining of mammals. Upon breakdown in the digestive system, the aglycone undergoes enterohepatic recirculation, which slows its elimination from the body (Catteuw et al., 2020). Additionally, findings from Fleck et al. (2017) in piglets strongly support the enterohepatic recirculation of ZEN, which could lead to prolonged retention of ZEN-14-G or ZEN in these tissues. Notably, ZEN demonstrated widespread tissue distribution, with detectable levels in all examined tissues. The mean tissue concentrations followed a consistent pattern: liver > glandular stomach > pectoral muscle. The tissue-specific distribution pattern of ZEN-14-G (with peak concentrations in the glandular stomach and a progressive temporal decline) suggests preferential absorption or retention in the gastric mucosa, potentially due to direct exposure from oral administration or localized metabolic activity. Secondary accumulation in the liver implies hepatic involvement in its processing, likely through portal circulation uptake or metabolic conversion (e.g., deglycosylation to ZEN). The minimal levels in pectoral muscles reflect limited systemic distribution, which may result from efficient hepatic clearance, poor muscle tissue vascularization, or low affinity for muscle cell transporters. These observations align with the physicochemical properties of ZEN-14-G (such as polarity and molecular size) and highlight key differences from its parent compound, ZEN, which typically shows broader tissue penetration. Further research should clarify whether gastric accumulation reflects passive diffusion or active transport mechanisms and whether hepatic detection represents intact ZEN-14-G or its metabolites.

## Conclusion

This study characterized the toxicokinetic profile of orally administered ZEN-14-G in broiler chicks, demonstrating its extensive conversion to ZEN in the liver and glandular stomach. Following rapid gastrointestinal absorption of ZEN-14-G, ZEN itself showed sustained accumulation at all measured time points. The persistence of ZEN, resulting from its slow elimination kinetics, suggests its potential as a robust biomarker for ZEN-14-G exposure. These findings significantly advance our understanding of modified mycotoxin metabolism in poultry and provide essential data for improving risk assessment models related to ZEN contamination in broiler production systems.

## CRediT authorship contribution statement

**Okasha Hamada:** Writing – review & editing, Writing – original draft, Visualization, Validation, Investigation. **Haojian Sun:** Validation, Methodology, Investigation. **Xue Pan:** Writing – original draft, Methodology, Data curation. **Decheng Suo:** Software, Methodology, Formal analysis. **Xia Fan:** Software, Methodology, Formal analysis. **Zhigang Song:** Writing – review & editing, Supervision, Project administration, Funding acquisition, Conceptualization.

## Disclosures

The authors declare that there are no known competing financial interests or personal relationships that could have influenced the conduct or outcomes of the research presented in this study.

## Appendix Supplementary materials

Image, application 1
