# Supplementary material for: Toxicokinetics and tissue-specific biotransformation of modified mycotoxin zearalenone-14-glucoside (ZEN-14-G) in broilers following oral administration
Source: Poult Sci. 2026 Jan 13;105(3):106445. doi: 10.1016/j.psj.2026.106445 (PMC12856181; doi:10.1016/j.psj.2026.106445)
Supplement: Supplementary file 1 [file mmc1.docx]

Table S1. Calibration plots of Zearalenone-14-glucoside and Zearalenone in plasma, liver, glandular stomach, and pectoral muscle

| **Items** | **Composition** | **slope** | **R2** | **Range (µg/L) (µg/kg)** | **LOD** | **LOQ** |
| --- | --- | --- | --- | --- | --- | --- |
| **Plasma** | **ZEN-14-G** | 1937 | 0.9998 | 1.25 – 100 | 1.17 | 3.53 |
|  | **ZEN** | 2485 | 0.9996 | 1.25 – 100 | 1.65 | 4.99 |
| **Liver** | **ZEN-14-G** | 1847 | 0.9991 | 1.25 – 100 | 2.52 | 7.36 |
|  | **ZEN** | 2237 | 0.9999 | 1.25 – 100 | 1.89 | 3.59 |
| **G. stomach** | **ZEN-14-G** | 2145 | 0.9999 | 1.25 – 100 | 0.76 | 2.30 |
|  | **ZEN** | 2010 | 0.9999 | 1.25 – 100 | 0.73 | 2.21 |
| **P. muscles** | **ZEN-14-G** | 2091 | 0.9997 | 1.25 – 100 | 1.49 | 4.52 |
|  | **ZEN** | 1975 | 0.9997 | 1.25 – 100 | 1.43 | 4.32 |

ZEN-14-G (zearalenone-14-glucoside), ZEN (zearalenone), G. stomach (glandular stomach), P. muscles (pectoral muscles), R² (R-squared), LOD (limit of detection), LOQ (limit of quantification). Plasma: µg/L; tissues: µg/kg

Table S2. Tissue residue levels of ZEN-14-G and ZEN in broiler chickens after single oral administration (0.5 mg/kg BW)

| **Time (h)** | **ZEN-14-G and ZEN (µg/kg)** | | | | | |
| --- | --- | --- | --- | --- | --- | --- |
|  | **Liver** | | **Glandular stomach** | | **Pectoral muscle** | |
|  | **ZEN-14-G** | **ZEN** | **ZEN-14-G** | **ZEN** | **ZEN-14-G** | **ZEN** |
| **0** | ND | ND | ND | ND | ND | ND |
| **30 min** | 0.648±0.345 | 12.037±3.31 | 34.239±7.517 | 4.103±0.895 | 0.6614±0.111 | 4.497±2.307 |
| **1h** | 0.339±0.171 | 5.307±2.705 | 35.904±18.277 | 2.955±1.274 | ND | 0.808±0.336 |
| **6h** | ND | 1.161±0.722 | 17.156±4.956 | 3.552±1.099 | ND | ND |
| **12h** | ND | 0.409±0.030 | 3.1644±1.500 | 0.872±0.499 | ND | ND |

ND= not detected
